# Selective detection of complex gas mixtures using point contacts: concept, method and tools

**DOI:** 10.3762/bjnano.11.146

**Published:** 2020-10-28

**Authors:** Alexander P Pospelov, Victor I Belan, Dmytro O Harbuz, Volodymyr L Vakula, Lyudmila V Kamarchuk, Yuliya V Volkova, Gennadii V Kamarchuk

**Affiliations:** 1 Department of Physical Chemistry, National Technical University “Kharkiv Polytechnic Institute”, 2 Kyrpychov Str., Kharkiv, 61002, Ukraine; 2 Department of Spectroscopy of Molecular Systems and Nanostructured Materials, B. Verkin Institute for Low Temperature Physics and Engineering, 47 Nauky Ave., Kharkiv, 61103, Ukraine; 3 Department of Pediatrics and Rehabilitation, SI “Institute for Children and Adolescents Health Care” of NAMS of Ukraine, 52-A Yuvileinyi Ave., Kharkiv, 61153, Ukraine; 4 Department of Pediatrics, V. Karazin Kharkiv National University, 4 Svobody Sq., Kharkiv, 61077, Ukraine; 5 Laboratory of Age Endocrinology and Metabolism, SI “Institute for Children and Adolescents Health Care” of NAMS of Ukraine, 52-A Yuvileinyi Ave., Kharkiv, 61153, Ukraine

**Keywords:** breath profile, cortisol, hormone detection, point contact, quantum sensor, selective detection, serotonin, Yanson point contacts

## Abstract

Of all modern nanosensors using the principle of measuring variations in electric conductance, point-contact sensors stand out in having a number of original sensor properties not manifested by their analogues. The nontrivial nature of point-contact sensors is based on the unique properties of Yanson point contacts used as the sensing elements. The quantum properties of Yanson point contacts enable the solution of some of the problems that could not be solved using conventional sensors measuring conductance. In the present paper, we demonstrate this by showing the potential of quantum point-contact sensors to selectively detect components of a gas mixture in real time. To demonstrate the high efficiency of the proposed approach, we analyze the human breath, which is the most complex of the currently known natural gas mixtures with extremely low concentrations of its components. Point-contact sensors allow us to obtain a spectroscopic profile of the mixture. This profile contains information about the complete set of energy interactions occurring in the point contact/breath system when the breath constituents adsorb to and desorb from the surface of the point-contact conduction channel. With this information we can unambiguously characterize the analyzed system, since knowing the energy parameters is key to successfully identifying and modeling the physicochemical properties of various quantum objects. Using the point-contact spectroscopic profile of a complex gas mixture it is possible to get a functional dependence of the concentration of particular breath components on the amplitude of the sensor output signal. To demonstrate the feasibility of the proposed approach, we analyze the point-contact profiles from the breath of several patients and compare them with the concentrations of serotonin and cortisol in the body of each patient. The obtained results demonstrate that the proposed methodology allows one to get an effective calibration function for a non-invasive analysis of the level of serotonin and cortisol in the human body using the point-contact breath test. The present study indicates some necessary prerequisites for the design of fast detection methods using differential sensor analysis in real time, which can be implemented in various areas of science and technology, among which medicine is one of the most important.

## Introduction

The functioning of devices comprising low-dimensional structures as basic elements depends on quantum effects, which play a crucial role in the unique properties of nanomaterials [1–2]. In order to achieve the maximum efficiency in using nanostructures, a comprehensive use of the fundamental scientific basis is necessary to open up opportunities for the creation of new devices and technologies. This approach to enlarging the variety of analytical tools includes using Yanson point contacts as the basic element of quantum sensor devices [3–4].

Of all modern nanosensors measuring a variation in electrical conductance, point-contact sensors have a number of unique properties. The nontrivial nature of point-contact sensors is based on the distinctive properties of the Yanson point contacts [4], used as the sensing elements. Spectral studies clearly demonstrate the fundamental difference between Yanson point contacts and traditional electrical contacts. The latter normally behave as bulk conductors of electric current without spectral properties. In contrast, Yanson point contacts display quantum properties that enable the direct measurement of the interaction between electrons and various quasiparticles, such as phonons or magnons [5–6], the observation of the spectral aspects of processes occurring in the superconducting state [7–8], and the extraction of spectral information from the electric noise [9]. Similar advantages arise in sensor studies. In this case, Yanson point contacts are responsible for the unique properties of point-contact sensors, which make them different from the existing analogues. In sensor studies, the quantum properties of Yanson point contacts solve the problems that could not be solved when analogues were used to measure the variation in electrical conductance. This can be exemplified by the new quantum mechanism of selective detection in gaseous and liquid media [10] and by the possibility of real-time detection of carcinogenic strains of *Helicobacter pylori* [11].

The original properties of point-contact sensors significantly contribute to their functionality and lay the foundation for a wide spectrum of new applications and solutions to new complex problems. In the present paper, we demonstrate the unique property of a quantum point-contact sensor to selectively detect components of a gas mixture in real time. The importance of using point-contact sensor devices to solve complex problems is highlighted by, at least, two facts. First, our analyzed medium, the human breath, is the most complex of the currently known natural gas mixtures. Second, the concentrations of the components that are quantitatively related to the detected substances are extremely low in the mixture.

The human breath is a complex biological medium, which contains more than 2000 components [12]. It has been widely studied by using a broad spectrum of techniques [13]. The main goal of these studies is to find peculiarities of the human metabolism that are typical of various diseases and to develop new noninvasive medical diagnostic methods. The functioning of the human body is accompanied by the biosynthesis of specific chemical substances. These substances become part of the exhaled breath and, as such, can be used as markers for breath characterization. Hence, it is important to detect single components of the exhaled breath for medical purposes.

Mass spectrometry, gas chromatography–mass spectrometry, as well as various optical spectroscopy techniques are successfully used to detect substances in the breath [14–20]. Yet, the high costs of the equipment and the need for highly skilled staff to operate and maintain it impede the large-scale implementation of these techniques in daily medical practice. From this point of view, it seems promising to use sensor devices to analyze the breath [21–23]. The main advantage of using sensors is that they are portable, inexpensive, and easy to use [24–25]. Gas sensors can detect breath components in extremely low concentrations and, thereby, they enable the development of medical diagnostic methods based on the concept of marker identification. However, this approach has a significant drawback since the processes happening in complex gaseous media are highly dynamic. Therefore, it negatively impacts the efficacy of medical diagnosis.

In the breath, there is a high possibility of interactions between the components, similarly to any multicomponent medium that contains substances of different chemical nature [26]. These interactions may eventually result in significant changes not only in the concentrations of the original components, but also in the chemical composition of the gas mixture. The breath contains oxidizing agents (e.g., nitrogen oxides, carbon oxide, and sulfur oxides), reducing agents (e.g., hydrogen sulfide, ammonia, mercaptans, and organic molecules), and other chemically active components that can participate in various chemical transformations. It is known, for example, that hydrochloric acid interacts almost instantaneously with ammonia to form NH_4_Cl. Therefore, there is a high probability that the composition of the human breath medium is transformed after it is formed in the human body and before it is released in the atmosphere. It should also be noted that the new compounds formed as a result of these interactions can be catalysts for new chemical reactions. This leads to a change in the original state of the breath, which may cause false-positive or false-negative results in medical diagnosis [27–28]. This problem has motivated the development of an alternative approach to analyze the breath, which is based on the inherent characteristics of the gas mixture. This new approach aims to obtain an integral portrait of the analyzed complex gas mixture, which is called the “breath profile” or the “breath fingerprint” [13]. Generally, the breath profile can be obtained by finding the complete composition of the breath, the concentration of its components, and the temporal dynamics of the changes in breath composition. In the case of a medium with more than 2000 components, this is a difficult task even for the most modern sensor devices. This problem is solved by using sensor devices known as “electronic noses” [29]. These modern systems can obtain up to a few dozens of points for the breath profile. But this is not enough to get a comprehensive portrait of the analyzed breath medium, which contains hundreds or thousands of components. As a consequence, there is a high probability of low reproducibility of the results obtained under different experimental conditions or in a series of independent experiments performed in different locations and over different periods of time.

It is clear that the process to obtain a complete dataset of a real breath profile at a specific time interval is extensive and time-consuming. In addition, it does not guarantee reliable results. To avoid these risks, new approaches must be developed. Quantum detection offers several possibilities to find and implement new mechanisms to selectively detect various substances. Therefore, it has the potential to become a breakthrough approach [4,10].

To implement the new quantum approach in sensor technology, new tools with unusual quantum properties need to be developed and applied. These new sensor devices can be based on Yanson point contacts [3–4]. Yanson point contacts are well known as the main instrument of Yanson point-contact spectroscopy [5–6]. They are quantum objects that are easy to produce and use. In addition, they are a source of unique information that is hard or even impossible to obtain with other methods. Yanson point contacts have a number of unique electrophysical properties, one of them being the nonequilibrium electron distribution function, which is formed in the current state of the point contact [30]. This function reflects the presence, in the area of a point contact, of a group of electrons in nonequilibrium states, which is formed as the electrons get accelerated by the electric field of the contact. The electric field is concentrated in the Yanson point contact area due to a specific electric drop in the potential in that area as the current flows through the electrode/point contact/electrode system. Upon crossing the area of the Yanson point contact, electrons are excited and transition to a nonequilibrium state. Then, the electrons gradually relax as they interact with quantum quasiparticle excitations and other objects. This process is fundamental for Yanson spectroscopy of electron–phonon interactions [30] and is responsible for the unique properties of point-contact sensors [4].

Point-contact sensors can detect single substances [31–32] and also obtain an integrated profile of a complex gas mixture [4,11]. This profile contains information about the complete set of energy interactions that take place in the point contact/ breath system. As a result, the obtained function can unambiguously characterize the analyzed system, since the knowledge of the energy parameters is the key to successfully identifying and modeling the physical–chemical properties of various quantum objects. In the present paper, by using the quantum properties of Yanson point contacts, we propose a new method that selectively detects the breath components based on the point-contact energy profile of this complex gas mixture.

## Experimental

In the present paper, we studied the breath of patients treated at the State Institution "Institute for Children and Adolescents Health Care at the National Academy of Medical Sciences of Ukraine", within the framework of the program that studies the fundamental mechanisms of disease development in the upper gastrointestinal tract. The development and testing of the method to detect the breath components involved patients in the age range between 12 and 18 years who had functional dyspepsia. The average age of the participants was 15.5 years. The study involved a total of 40 participants, among which 22 were male and 18 were female patients. Patients younger than 12 and older than 18 years old, or who had at least one of the following symptoms, such as acute diseases, fever, unintentional weight loss, blood vomiting, melena, anemia, leukocytosis, or destructive changes in the mucosa of the upper digestive tract were excluded from the studies.

The clinical diagnosis was made based on the medical history of the patients, complaints of the patients, and results obtained from physical examination. Special attention was paid to the identification and detailing of clinical symptoms based on the medical history, the presence of a family history of digestive system lesions, and disease duration, in accordance with the Rome IV criteria [33]. All patients received a full medical examination identical to the previously described procedures [11].

The breath profile was registered using point-contact sensor matrices based on tetracyanoquinodimethane (TCNQ) compounds [34] in accordance with the method we developed and verified in our earlier medical studies [11,35]. A point-contact sensor matrix is a structure consisting of a large number of mechanically stable Yanson point contacts. These contacts correspond to point contacts produced using the Chubov technique [36], which is the most efficient technique used in Yanson point-contact spectroscopy [4–5]. The density of the primary nanostructured sensing elements in the sensor matrix was between 5 × 10^4^ and 6 × 10^4^ Yanson point contacts per square millimeter of the observable surface.

The mesoscopic point-contact sensor matrix has a small integrated source of energy, which is manufactured with a special technique during the synthesis of this nanostructure. The production of the energy source and the matrix as a whole is based on electrochemical processes described previously [34,37–38]. The applied technology guaranteed high reliability and longevity to the sensor elements. Point-contact sensor matrices can function autonomously without an external energy source for more than a year even under field conditions [11].

The portable sensor system used for this study was developed by us earlier and consists of the following main elements [39]: a holder with an electrical connector for the sensor, which also has all the necessary elements for communication and transmission of the sensor signal to the recorder; a programmed recorder, which can not only register the sensor signal and transmit it to a computer, but also function as a signal amplifier if necessary; a computer with an original software (developed at the B. Verkin Institute for Low Temperature Physics and Engineering of the National Academy of Sciences of Ukraine) used for storing information and performing real-time data analysis.

The process of obtaining the breath profile starts when the sensor is mounted on the electrical connector at the upper end of the holder, before the measurements are taken. Then, a disposable hood, in the form of a removable cone-shaped tube, used for the formation of a cell containing the sensor, is attached to the upper end of the holder. During the measurement, this cell is kept inside the mouth of the patient for 1 min. Thus, the sensor is directly exposed to the breath of the patient. Any influence from the external environment can be excluded. The optimal exposure time was experimentally selected based on previous studies [11,35,40]. It corresponds to the period of time required to have all breath components interact with the sensing elements of the point-contact sensor matrix. When the exposure is over, the sensor cell is removed from the mouth and the relaxation of the sensor in the ambient atmospheric environment begins. The relaxation time depends on the composition of the breath and usually is in the range of 1–3 min. Thus, the whole process of registering the breath profile occurs in real time. After the relaxation, the sensor is ready for the next measurement. To increase the reproducibility and reliability of the obtained results, we made three consecutive measurements for each patient. According to the methodology we developed earlier for obtaining breath profiles [11,35], the measurements must be taken after the patient fasted overnight. On the day of the experiment, the patients did not take any medication. The fulfilment of these requirements guarantees the maximum exclusion of any side factors and effects that could alter the characteristic breath profile of a patient.

A heating of the sensor in order to prevent condensation of the exhaled breath inside the sensor holder was not performed in our studies since it is the condensate from the exhaled gas that activates the sensing element. A condensate film formed on the gas-sensing surface switches on the autonomous power supply of the sensing element, enabling the observation and measurement of the dynamics of resistance variation during the exposure and relaxation periods. This is due to the fact that, in the process of exposure, components of the exhaled breath diffuse through the condensate film to the surfaces of the conduction channels of the point-contact matrix. This impacts the resistance characteristics of each one of the point-contact nanostructures and, as a result, the resistance of the whole matrix.

The metabolic profile of a patient is registered by measuring the dependence of the electrical conductance of the point-contact sensor matrix with time. This dependence is called the response curve. The complete metabolic profile of the patient is obtained through the response curves measured during the exposure and relaxation of the sensor.

A set of 49 sensors was manufactured for the study. To avoid the influence of any variation in some of the electrical parameters of the sensor on the experimental results, we selected samples with the most similar parameters. All manufactured samples were tested using the breath media from healthy participants as a control. Cluster analysis of the testing results using the criteria we developed earlier [41] was performed to select sensors with the most similar parameters. As a result of these procedures, a set of 25 sensors was selected in which any pair of sensing elements had a very similar response to the analyzed gas mixture [34,41].

To test the proposed new method in which single components of a complex gas mixture are selectively detected using point-contact sensors, serotonin and cortisol were chosen as the substances to be detected. The concentration of these substances was obtained from the breath of the patients during the medical studies. These substances were selected because of the important role they play in the metabolic processes of the human body.

Serotonin (5-hydroxytryptamine, C_10_H_12_N_2_O) has several biological functions [42–43]. It plays an important role in regulating human psycho-emotional states [44–45]. The serotonin concentration increases during euphoria and decreases during depression and this is why serotonin is often called the “hormone of happiness”. Serotonin participates in the physiological regulation of various functions of the digestive organs, including gastric secretion and motility [46–48]. Between 60% and 90% of all serotonin generated in the human body is produced in the digestive tract [49], which is where this amine becomes part of the exhaled breath.

Cortisol (11β,17α,21-trihydroxypregn-4-ene-3,20-dione, C_21_H_30_O_5_) is a biologically active hormone produced by the adrenal cortex. It belongs to the class of glucocorticoid hormones [50] and it controls several functions in the human organism [51]. For example, it participates in the carbohydrate, lipid, and protein metabolism, is responsible for the energy conservation in the organism, and affects the synthesis of cellular enzymes. Cortisol generates the defense reactions of the organism against external threats and stressful situations [52]; therefore, it is called the “stress hormone”. In the case of heavy stress, cortisol changes the muscle activity by decreasing their glucose consumption and stimulating its arrival to other organs. Cortisol prevents the secretion of substances in the organism that can cause inflammation [53]. At the same time, a high level of cortisol inhibits the immune system and worsens the ability of the organism to resist against infections and other harmful factors [54].

A comprehensive study of how the concentrations of serotonin and cortisol vary in the human body can promote a better understanding of various disease mechanisms and help to find new approaches for improving medical treatment. Overall, this contributes to the development of new noninvasive tests for the detection of these hormones.

The concentration of serotonin in the blood was determined by the fluorometric method [55–56] whereas the concentration of cortisol in the blood serum was determined via the enzyme-linked immunoassay using the reagents produced by the NPL Granum company (Kharkiv, Ukraine).

Statistical calculations were made using the WEKA data mining software [57]. Additional control and verification of the results were performed with the SPSS statistical package [58]. The data were processed statistically using the standard parametric and nonparametric methods [59].

## Ethics

The research protocol was approved by the Institutional Review Board and the Ethical Committee of the State Institution "Institute for Children and Adolescents Health Care at the National Academy of Medical Sciences of Ukraine". All the patients gave their written consent to participate in the study.

## Results and Discussion

The idea of the method proposed here is based on the fact that any spectrum contains the energy information about the components of a system and their interactions. This paradigm is actively used as the basis of many research methods and techniques (e.g., optical spectroscopy). The application of the energy approach to solve practical problems is often limited by the complexity of the systems under study and the lack of simple and reliable tools to measure and analyze processes. The implementation of innovative mechanisms based on the quantum properties of objects and the usage of quantum tools and methods provide new unlimited opportunities for the development of technologies based on the principles of data collection and analysis of energy.

Earlier, we proposed to use the information about energy processes occurring in the breath for its detection and analysis [11]. The idea was feasible and could be implemented using appropriate tools that met the basic requirements for modern sensor technology, including simplicity, high efficiency, portability, low cost, and real-time detection. All of these criteria are met by modern nanosensors created on the basis of Yanson point contacts. A Yanson point contact is a unique quantum object and the main instrument for Yanson point-contact spectroscopy. The operating principle of Yanson point-contact spectroscopy is based on measuring the interaction between electrons and other quantum quasiparticle excitations using point contacts. This was clearly demonstrated for the first time by Yanson while studying electron–phonon interactions in metals [60]. When a current flows through Yanson point contacts, a unique condition for the manifestation of the quantum properties of these objects arises. These properties are essential for Yanson point-contact spectroscopy [6] and are used to create a new generation of advanced nanosensors [4].

The Yanson point contact itself is the simplest electrophysical structure and it can be represented as an electrode/point contact/electrode system. In the electric current mode, in which this system is usually studied, the decrease in the voltage applied to the system occurs exclusively in the area of the point contact [30]. This allows for the concentration of the electric field in a small region, which serves as an additional energy source for the electrons passing through the contact, leading to acceleration and subsequent transition to a nonequilibrium state. As a result, a nonequilibrium electron distribution function is formed in the contact area [30]. The electron relaxation upon interaction with other groups of quasiparticles leads to the manifestation of the energy parameters of this interaction in the electrical characteristics of the point contacts. Due to this phenomenon, the spectrum of the electron–phonon interaction in metals [5], superconductors [8,61], and even in more complex compounds such as organic conductors [62] can be easily obtained. The point-contact spectrum of the electron–phonon interaction contains the information about the characteristic parameters of the phonon system of a material, which is difficult or even impossible to obtain using other methods.

**Figure 1:**
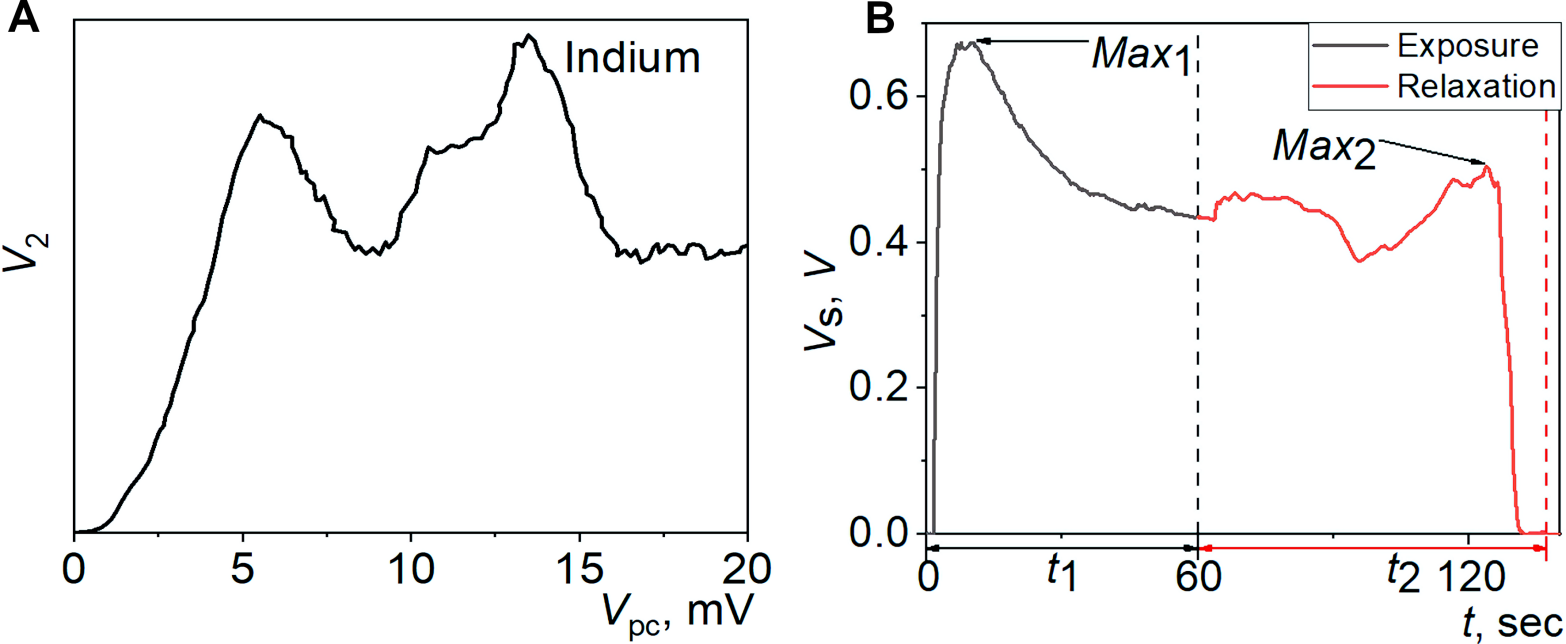
(a) Spectrum of the electron–phonon interaction in indium obtained using Yanson point contacts. *V*_2_ is the second derivative of the current–voltage characteristic of the point contact and *V*_pc_ is the decrease in contact voltage. (b) A typical time dependence of the point-contact sensor conductance based on TCNQ compounds as a result of its interaction with the human breath (i.e., the breath point-contact sensor profile). *V*_s_ is the voltage decrease that occurs in the sensor, *t* is the time, *t*_1_ is the exposure time, and *t*_2_ is the relaxation time.

In order to understand our proposed approach to analyze the human breath, one should first consider the point-contact spectrum of the electron–phonon interaction in indium [63] (Figure 1a). The abscissa directly characterizes the energy of the phonons that interact with the electrons, which in turn obtain an excess energy of the order of *eV_pc_* when accelerated by the electric field in the Yanson point contact. Here *e* is the electron charge and *V_pc_* is the voltage applied to the contact. Thus, each section of the curve reflects the energy parameters of a certain group of phonons. This fact allows, for example, the determination of phonon groups with energy values that are characteristic of various types of atomic vibrations in a solid. The ordinate of the point contact-spectrum characterizes the phonon concentration in a certain section of the spectrum and reflects the amplitude of the density function of the phonon states.

The operation of point-contact sensors is in line with the principles of Yanson point-contact spectroscopy [3,6]. Point-contact sensors exhibit quantum properties during the detection of various objects and enable new detection mechanisms [4,10]. The quantum properties of the point-contact sensors enable the measurement of the breath spectral profiles, which contain comprehensive information about this complex gas medium [11]. One representative point-contact breath spectral profile obtained in the present study is shown in Figure 1b. As already mentioned, the full point-contact sensor profile of the breath consists of two parts obtained during exposure and relaxation. In this profile, the exposure curve encompasses the time range between 0 and 60 s whereas the range above 60 s corresponds to the relaxation curve. The point-contact sensor breath profile has a nonmonotonic spectral character with a number of nonlinearities, maxima, and minima. Unlike other nanosensors and conventional sensors based on the principle of changing electrical conductance, point-contact sensors are able to explicitly display energy interactions and their features in the breath profile. This is possible because, in Yanson point contacts, heat generation and nonlinear electric current processes in the contact caused by scattering of electrons in a nonequilibrium state are spatially separated [30]. The separation of nonlinear electric current phenomena from other interactions allows us to directly observe their energy component as a result of the interactions in the point contact/breath system. This property of the Yanson point contact arises from the large mean free path of the electrons in the contact, which allows for the manifestation of all thermal effects outside the contact. Thus, the interaction-related nonlinear changes in the point-contact conductance result only from scattering processes of electrons in a nonequilibrium state [30]. In addition, the voltage sweep in the contact ranges widely and so does the excess energy of nonequilibrium electrons, which they can transfer as a result of the interaction. This allows us to effectively influence the processes of adsorption and desorption and directly measure their energy parameters.

Thus, the operation mechanism of the point-contact sensor and the measurement of the energy parameters of the components of the analyzed system can be understood as follows. Depending on the nature of a given molecule, the molecule can be characterized by a certain energy of interaction with the gas-sensing matrix material (i.e., the adsorption energy). Any adsorption event in the Yanson point contact entails a redistribution of the electron density in the conduction channel of the point contact, the scattering of the conduction electrons in a nonequilibrium state on adsorbed atoms, the transfer of the excess energy of electrons to the adsorbed atoms followed by their desorption, and the relaxation of the nonequilibrium distribution function of electrons in the contact, which results in a change in the point-contact resistance. These changes are displayed in the dependencies of the Yanson point contact parameters. In the case of classical Yanson point-contact spectroscopy studies of electron–phonon interaction in metals, the energy-related processes are described by the current–voltage characteristic of the Yanson point contacts and its derivatives. In our study of the exhaled breath, the energy-related processes can be seen as the temporal variations of the electric conductance of the point contact and plotted as a spectral profile of the exhaled breath. The fundamental properties of Yanson point contacts enable the measurement of the electric conductance variations when they are equivalent to at least one conductance quantum. This guarantees an extremely high sensitivity of the point-contact sensors and the measurement of minute energy perturbations occurring during the interaction of the breath components with the point-contact conduction channel. Therefore, the nature of the molecule is revealed through the energy parameters of the molecule.

In the initial state, the gas-sensitive surface of the sensor is in equilibrium with the surrounding atmosphere. Experiments show that the nature of the gas-sensitive material provides a reversible physical adsorption under the action of substances that prevail in the environment. When there are gaseous analyte substances near to the gas-sensitive surface, they occupy a certain portion of the active centers as a result of a competing adsorption. This process changes the resistance of the point-contact array. Therefore, there is no need to provide special storage conditions for the sensor before the measurements. The nature of the active centers is such that, between measurements, the atmospheric nitrogen is able to completely block them and preserve the gas-sensitive layer, neutralizing the influence even of active oxidizing agents such as oxygen.

When the sensor is placed in the oral cavity, the nature and thermodynamic parameters of the gas-sensitive surface/gaseous-environment system change dramatically. The temperature and humidity rises and the chemical composition of the gaseous medium changes. With an increase in humidity and temperature, the galvanic element, which is integrated in the point-contact gas-sensitive array and generates the electric field, is activated. The field initiates quantum-energy effects in the bulk and on the surface of the conduction channels of Yanson point contacts. At the active centers, molecules of metabolic products and their derivatives replace the nitrogen molecules. Under these conditions, the system transitions to a new quasi-equilibrium state. The events occurring in this case correspond to the quantum dissipation of energy, which is measured as a voltage change in the sensor element by a special device. Since the relaxation process lasts for about a minute, it can easily be recorded as an equidistant time series. We consider this series as a sensor response to the breath medium during the exposure period in the oral cavity (or simply during the exposure period). The next phase of the measurements consists of recording the voltage in the sensor when it comes into contact with the original atmosphere after the exposure in the oral cavity is over. This process is opposite to the previous one and it is caused by the desorption of the breath components and the return of atmospheric nitrogen molecules to the active centers. It is also initiated by the electric field of the galvanic cell integrated into the point-contact array. Yet, in this case, due to a decrease in humidity and temperature, the field also decreases. The reverse process, which is essentially the relaxation step, usually lasts between 1 and 3 min. The exposure and relaxation processes are measured as an equidistant time series which represents the sensor metabolic profile of the patient (Figure 1b).

The adsorption–desorption processes on the active surface of the sensor are eventually reversible and are characterized by a significant hysteresis. This complex nature of the response unfolds broad prospects for identifying the specific metabolism of each patient. Therefore, using the sensor in medical diagnostics is an efficient tool to analyze the breath medium. Some progress has already been made in this area. In the complex response configuration, the so-called “characteristic parameters” have been already distinguished in a few published studies [3,35,40]. Regular individual differences measured in the breath of some of the patients may be caused by metabolic peculiarities and may indicate the presence of a certain pathology. The characteristic parameters were analyzed considering the history and clinical parameters of the examined patients (i.e., results of clinical, laboratorial, and instrumental examinations). Next, a search for relevant correlations between the analyzed parameters of the patient and the characteristics of their breath was made. The obtained correlations were used as criteria for determining the deviation of the sensor curve response in patients with a pathology in comparison with patients with a normal metabolism. The detected deviation of the response curve of the sensor made it possible to identify one or more pathologies or to assign patients to certain risk groups. This approach was quite fruitful and enabled the development of the scientific basis for breath tests in early non-invasive diagnosis of erosive-ulcerative lesions of the gastrointestinal tract based on the detection in the breath of ill patients of waste products from the bacterium *Helicobacter pylori*, including cytotoxic strains of this microorganism.

However, it should be noted that further improvement of analytical procedures involves lowering the sensitivity threshold of the sensor in relation to a wider list of controlled substances and their derivatives. This, in turn, requires a finer and more detailed analysis of the features of the response curve configuration. A reliable tool for the precise differentiation of a complex response curve can be a systematic approach to the synthesis of its characteristic elements and the search for singularities associated with the target object.

One of the directions of the systematic approach can be related to the analysis of the level of correlation of the sensor output parameter (voltage) with the target detection parameter (marker concentration in the breath) during the time of the analysis (exposure period plus relaxation period). This analysis is convenient since a complex response curve reflects the totality of interactions of a complex gaseous medium including the target substance with the gas-sensitive array. The higher the correlation level in a certain period, the higher the probability of the marker (or derivatives of its interaction with certain components of the breath) to give a signal in the response curve. These marked sections have a physical meaning: Gas atoms adsorbed on the conduction channel of the Yanson point contact create areas with distortions in the crystal structure of the contact material. This leads to a shortening of the mean free path of electrons in the contact area and increases the probability of scattering of nonequilibrium electrons at the sites of the adsorbed atoms. As a result of the scattering, the excess energy of the electron is transferred to the adsorbed atoms, which subsequently desorb. Upon transferring the excess energy to the gas atoms the electrons return to their equilibrium state. In turn, the gas atoms, upon receiving enough energy, desorb from the surface of the Yanson point contact. The process proceeds in a real-time regime enabling the measurement of the energy profile of a complex gaseous medium (e.g., the human breath). The result of the interaction is displayed on the time axis of the sensor response curve, since the duration of the interaction actually reflects the amount of energy transferred. Thus, by analogy with the point-contact spectra of the electron–phonon interaction, the point-contact profile of the breath contains information about the energy parameters of the atoms and molecules adsorbed on the surface of the Yanson point-contact conduction channel.

The target substance or the derivatives formed during its interaction with the components of a complex gaseous medium can change the resistance of the point-contact array when the galvanic cell creating the electric field is in a certain energy state. This, in turn, happens due to the peculiarities of the electron transfer through the conduction channel of the point contact viewed as the elementary unit of the gas-sensitive array. This allows us to propose a method for detecting individual components of a complex gaseous medium by determining the section of the curve of the spectral sensor profile that corresponds to the energy of interaction between the conduction electrons and these components.

The starting point of the proposed strategy for the analysis of the metabolic profile of the patients was to obtain the dependence of the correlation coefficient of the studied component of the breath with the level of the sensor output signal during the measurement. It was assumed that all points with a deviation of less than 2% from the maximum value can be used as the data in parallel experiments for obtaining the dependence of the response voltage on the concentration of a specific target substance. The dependence of the response voltage, for a period of maximum correlation with the analyte concentration, in a studied patient group allows us to obtain a linear regression equation. This equation can serve as a characteristic calibration curve for determining the concentration of the target component of a complex gaseous medium using a sensor.

By applying the above strategy to determine the concentration of hormones in the human body with the help of a sensor, we experimentally show the possibility of detecting singular characteristic sections of a complex response curve of a point-contact nanosensor upon breath exposure. To demonstrate the operability of the proposed approach, we analyze the point-contact breath profiles of the patients (the so-called metabolic profiles in the form of response curves of the point-contact nanosensor) and compare them with the values of serotonin and cortisol in the body of each patient.

To get the primary data, we obtained respiratory tests results from 16 patients with a known level of serotonin concentration in their blood (group 1) and from 16 patients with a known level of cortisol concentration in their blood serum (group 2). To be able to employ the spectral approach we proposed to detect the components of complex gas mixtures using point-contact sensors. For this we needed to identify the sections of the sensor response curves that were related to the metabolic profiles of the patients and were most informative regarding the concentration of the targeted hormone. To do this, we presented the sensor response in a time series form with a step of 0.5 s. Next, the temporal dependences of the correlation coefficient between the response voltage and the hormone concentration for groups 1 and 2 were determined. The linear correlation coefficient (i.e., the Pearson correlation coefficient) *r* between the response voltage *V* at a given time and the hormone concentration *C* was calculated using the following relation:

[1] $$r=\frac{{\mathrm{cov}}_{VC}}{\sigma_{V}\sigma_{C}}=\frac{\sum_{j=1}^{n}\left(V_{j}-\bar{V}\right)\left(C_{j}-\bar{C}\right)}{\sqrt{\sum_{j=1}^{n}{\left(V_{j}-\bar{V}\right)}^{2}\;\sum_{j=1}^{n}{\left(C_{j}-\bar{C}\right)}^{2}}},$$

where cov*_VC_* is the covariance of the values *V* and *C*, σ*_V_* is the root-mean-square deviation of *V*, σ*_C_* is the root-mean-square deviation of *C*, *V_j_* is the response voltage at a given time for the *j*-th patient (out of *n* patients), and *C_j_* is the hormone concentration of the *j*-th patient. 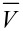 and 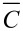 (see Equation 2) are the mean values of the respective samplings:

[2] $$\bar{V}=\frac{1}{n}\sum_{j=1}^{n}V_{j},\;\bar{C}=\frac{1}{n}\sum_{j=1}^{n}C_{j}.$$

**Figure 2:**
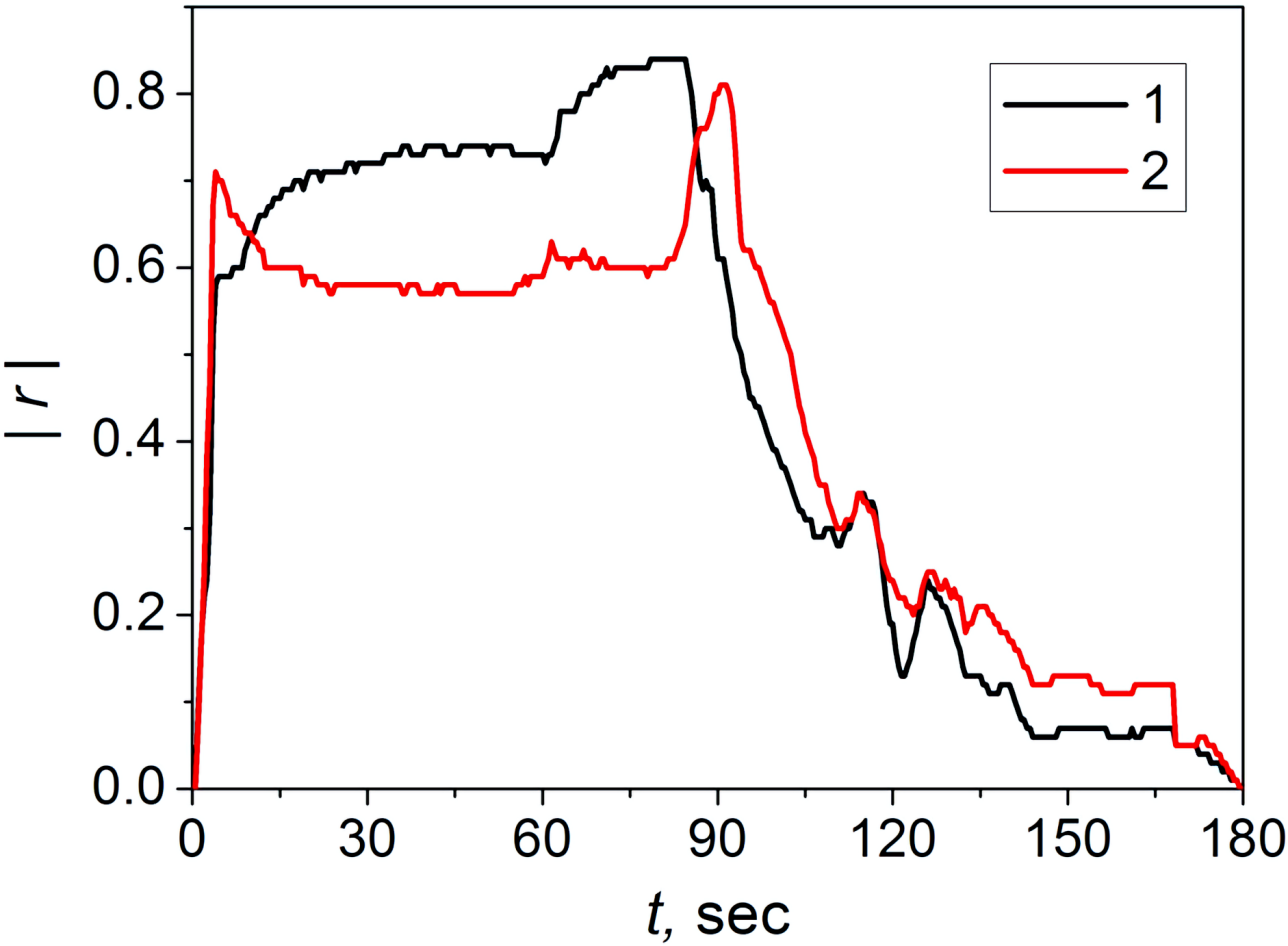
Temporal dependence of the absolute values of the correlation coefficient |*r*|. Here, *r* describes the correlation between the response voltage values of the breath tests of the patients and the concentration of serotonin (1, black curve) and cortisol (2, red curve) according to the medical tests.

The obtained correlation coefficients *r* were used for plotting the temporal dependencies of the absolute values |*r*| for the groups 1 and 2 (Figure 2).

The highest negative correlation for serotonin is observed in the range of 70–85 s and for cortisol in the range of 89–92 s. These time intervals of the measurements represent the period in which the hormones and the products of their interactions with other components of the breath are able to significantly affect the physicochemical state of the point-contact array and change the resistive characteristics of the gas-sensitive elements. The time range in which the dependence shown in Figure 2 was calculated is limited by the maximum time of the breath test. However, the total exposure time and relaxation time of the majority of the breath tests did not reach 180 s and the missing points of the response curves were assigned zero values.

**Figure 3:**
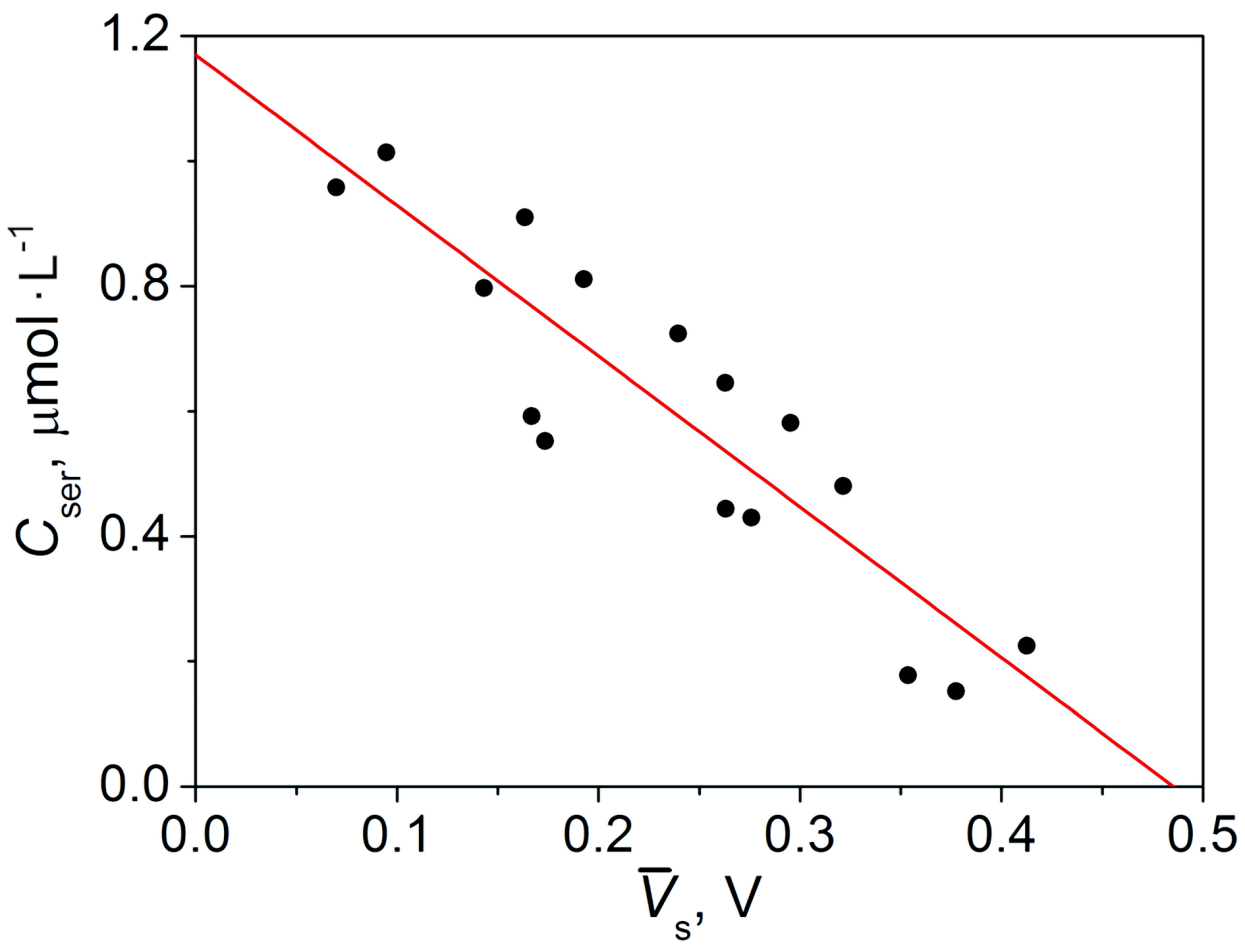
Dependence of the analytical concentration of serotonin *C*_ser_ on the average response voltage 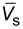 in the area of the maximum correlation. Black dots correspond to the data from the medical tests and the red line is the result of the linear approximation, given by Equation 3.

Thus, the characteristic time interval to estimate the level of serotonin in the blood of a patient via a breath test is in the range of 70–85 s. The values of the average voltage 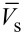 of the metabolic profile of a particular patient during this interval (Figure 2) and the concentration of serotonin *C*_ser_ in the blood uniquely determine the position of the points in the graph of response voltage as a function of the concentration (Figure 3). Since there is no information a priori about the nature of the relationship between the concentration of serotonin and the voltage of the sensor, it is reasonable to use a linear approximation and draw a straight line through the obtained points using the least-squares method:

[3] $$C_{\text{ser}}[\mu \text{mol}\cdot {\text{L}}^{-1}]=1.17-2.41\times {\bar{V}}_{\text{s}}.$$

This is a characteristic calibration line for estimating the serotonin concentration using the sensor method based on the breath test. Statistical analysis showed that the standard deviation of the medical analysis data from the approximation line does not exceed 0.115 μmol·L^−1^. Thus, with a 95% probability, the confidence interval is 0.230 μmol·L^−1^.

The model for determining the serotonin concentration (Equation 3) was verified by adding relevant results of the breath tests of four new patients from whom the serotonin content in the blood was known (Table 1).

**Table 1:** Verification of the sensor model for the determination of serotonin concentration.

Patient number	Average response voltage 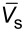 in the area of maximum correlation (V)	Serotonin concentration, *C*_ser_ (μmol·L^−1^)		

		Analysis data	Estimation from model (Equation 3)	Model error

1	0.253397	0.394	0.559	−0.165
2	0.263031	0.451	0.536	−0.085
3	0.281277	0.585	0.492	0.093
4	0.170466	0.733	0.759	−0.026

For group 2 (comprising of 16 people), in addition to the results of the breath tests, the cortisol content in the blood serum of the patients was also known. The correlation analysis of the cortisol metabolic profile showed that, in this case, the section of the maximum correlation is in the range of 89–92 s (Figure 2).

The dependence of the concentration of cortisol *C*_cor_ on the average voltage 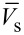 in the area of maximum correlation can be approximated by the linear equation:

[4] $${С}_{\text{cor}}[\text{nmol}\cdot {\text{L}}^{-1}]=765.54-1612.32\times {\bar{V}}_{\text{s}}.$$

**Figure 4:**
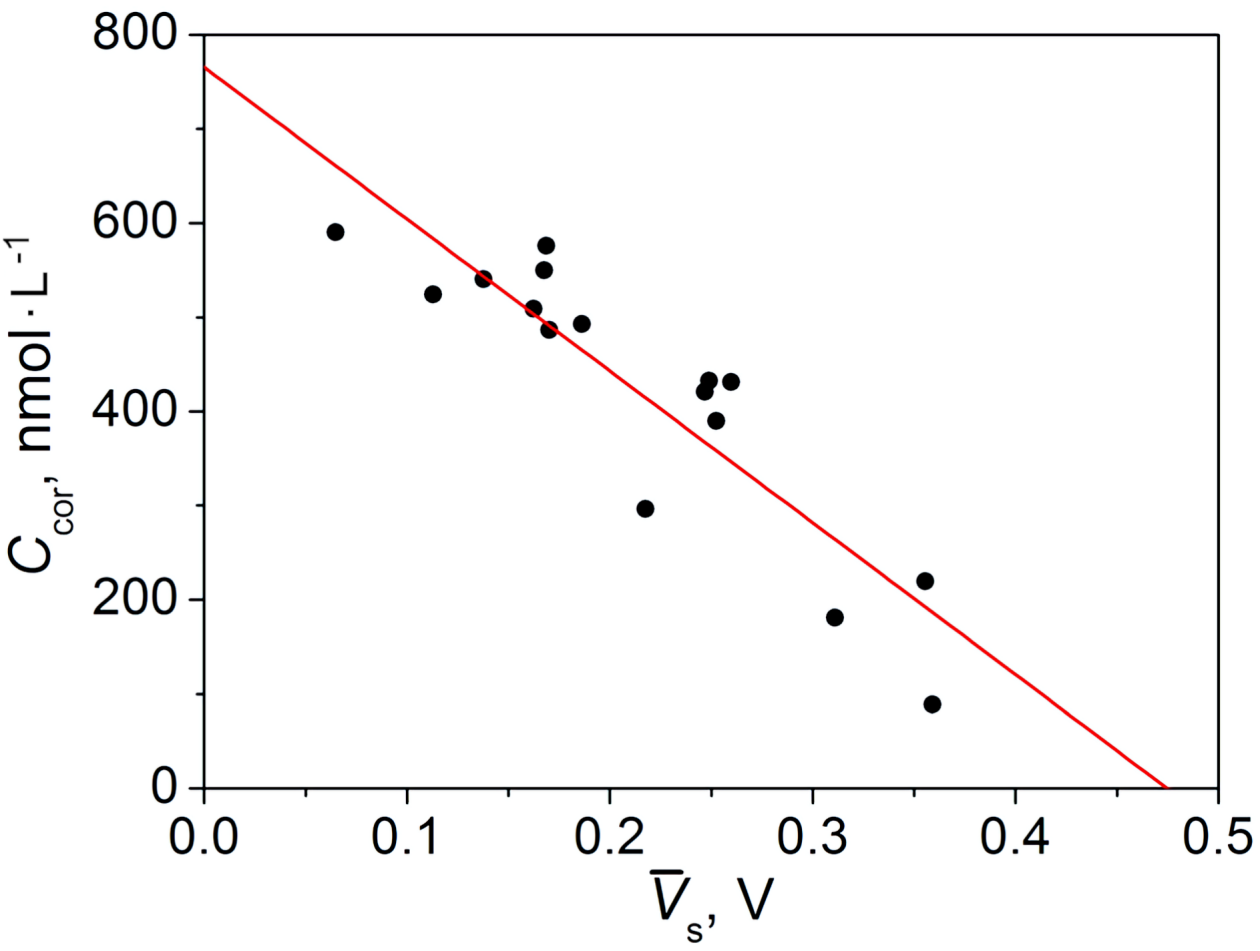
Dependence of the analytical concentration of cortisol *C*_cor_ on the average response voltage 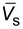 in the area of the maximum correlation. Black dots correspond to the data from the medical tests and the red line is the result of the linear approximation, given by Equation 4.

The results of this approximation are presented in Figure 4.

Statistical analysis showed that the standard deviation of the medical analysis data from the approximation line does not exceed 61.8 nmol·L^−1^. Thus, with a 95% probability, the confidence interval is 123.6 nmol·L^−1^. The verification of the model (Equation 4) for the determination of the cortisol concentration was carried out similarly to the verification of the model (Equation 3) for the determination of the serotonin concentration. Again, for four new patients with a known cortisol concentration in the blood serum were used to test the model (Table 2).

**Table 2:** Verification of the sensor model for the determination of cortisol concentration.

Patient number	Average response voltage 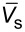 in the area of maximum correlation (V)	Cortisol concentration *C*_cor_ (nmol·L^−1^)		

		Analysis data	Estimation from model (Equation 4)	Model error

1	0.365943	166.6	175.5	−8.9
2	0.256083	300.0	352.7	−52.7
3	0.156115	498.8	513.8	−15.0
4	0.165320	570.3	499.0	71.3

The verification of the linear mathematical models, given by Equation 3 and Equation 4, showed that all the sensor analysis results are within the corresponding confidence intervals. This evidenced that the statistical calculation was appropriately done and the models can be used to estimate the concentration of hormones using breath tests. The obtained results demonstrate that the proposed mathematical models for obtaining the dependence of the concentration of the target substance on the output signal parameters of a quantum sensor yields an effective calibration function for a noninvasive analysis of the serotonin and cortisol levels in the human body using the breath test.

The results of the study clearly demonstrate the indisputable advantages of using nanotechnology in the field of sensor technology to develop and improve environmental-monitoring tools. The level at which the components of the analyzed complex gaseous medium were identified and the sensitivity of this detection, previously unattainable by conventional sensors, have become possible due to the in-depth differentiation of the gas-sensitive surface at the atomic level, under laboratory conditions. For the first time it became possible to analyze the human hormonal background using the breath, which is the most convenient biological material. With the hormones serotonin and cortisol used as examples, it was shown that concentrations of these substances can be monitored in real time, whereas in the case of conventional medical analysis it might take hours or even days.

Special attention should be paid to the unprecedented sensitivity of the developed point-contact nanosensor element. It should be reinforced that the studied analytes are not gaseous substances that can be directly found in the breath. Not exceeding a fraction of micromoles per liter in the blood, these molecules “transmit” the information about their quantity through a chain of mediators (transmitters), the last of which is recognized by the surfaces of the atomic structures of the gas-sensitive array (i.e., the Yanson point contacts). As a result, we can quantitatively estimate, with a probability of 95%, the stress level in the body. Wide prospects are opening for more detailed studies regarding the dynamics of the emotional states when various factors simultaneously affect the state of the organism, and our preliminary investigation confirms that. Due to the instability of the hormonal molecules, the validity of the conclusions and medical reports made based on the analysis from data sets taken during a long measurement period are being contested. Therefore, performing express tests using point-contact nanosensors for the observation of hormone concentration profiles in a dynamic manner is of undeniable importance.

The present study proposes a sequence of procedures to unravel the correlation between the concentration of a particular hormone and the level of the output signal. This dependence can be used to estimate the hormonal background of the patient using the data from the sensor analysis of the breath medium. The establishment of this correlation was possible due to the unique point-contact nanosensor with an ultrahigh surface density of information recognition elements in the form of Yanson point contacts. As a result, the characteristic output of this sensor is a time series of the complex profile. A systematic approach to decode the information coming from the internal organs of a human body allowed us to develop a universal approach to analyze a wide spectrum of breath components.

The presented study shows that a combination of advanced techniques based on Yanson point contacts, accessible material base, and modern analysis tools creates a robust and fast detection method in which the real-time differential sensor analysis can be widely applied in various areas of science and technology, especially medicine.
